# Analysis of the spatial distribution of the serve and the type of serve-return in elite table tennis. Sex differences

**DOI:** 10.3389/fpsyg.2023.1243135

**Published:** 2023-08-11

**Authors:** Francisco Pradas, Víctor Toro-Román, Carlos Castellar, Luis Carrasco

**Affiliations:** ^1^ENFYRED Research Group, Faculty of Health and Sports Sciences, University of Zaragoza, Huesca, Spain; ^2^Faculty of Sport Sciences, University of Extremadura, Cáceres, Spain; ^3^Faculty of Education, University of Sevilla, Sevilla, Spain

**Keywords:** racket sports, technique, strokes, game analysis, server, receiver

## Abstract

Serve and return of the serve are among the most critical technical-tactical factors influencing performance in table tennis (TT). The present study aimed to analyse sex differences in the spatial distribution of serve and serve-return in elite TT players. A total of 48 elite TT players (men: *n* = 24; women: *n* = 24) participated in the investigation. A total of 24 matches were recorded and examined, analysing 1,177 plays and 5,319 strokes in the men’s competition and 950 games and 5,097 strokes in the women’s competition. Technical actions were analysed using an observation tool validated by two expert TT coach with a high level of agreement (*K* > 0.80). Men distributed their serves over zones 1 and 2 of the table, while women more frequently selected zone 5 (*p* < 0.05). In men, 30.1% of the serves were near the net, 63.6% in the middle zone of the table and 6.2% in the end zone of the table, while in women, these values were 10.9%, 67.2%, and 21.8%, respectively. As for the technique of the serve-return depending on the zone of the service, in men the action of the cut from zone 1 and 2 predominated, while in women this technique was more frequent from zone 5 (*p* < 0.05). On the other hand, the serve-return with the flip technique in zone 1, 3 and 4 was more frequent in men (*p* < 0.05). As for the return of the serve with the topspin technique, there were differences in zone 3, being more frequent in men (*p* < 0.05). Finally, the serve return with cut and defensive serve-return techniques in zones 3, 4, 5, and 6 were more frequent in women (*p* < 0.05). There are differences between sexes in service and return of serve actions during a match in elite players.

## 1. Introduction

Table tennis (TT) has significantly transformed the rules and playing materials in the last two decades. These modifications have led to a more modern and dynamic TT (Pradas et al., 2021; De la Torre et al., 2022). The TT game is played on a rectangular table 2.74 m long by 1.52 m wide, 76 cm above the ground and separated in the middle by a net 15.25 cm high (Kawazoe and Suzuki, 2004). Considering the reduced dimensions of the playing surface, TT is characterised by developing a high-speed game dynamic, where intermittent physical efforts predominate, alternating short periods of rest with short but very high intensity and explosive efforts (Zagatto et al., 2010). In this sense, TT is considered one of the fastest sports in speed of play (Pradas et al., 2022).

In racket sports, the analysis of the game actions performed during competition has been the subject of essential studies as they are considered one of the most relevant aspects of performance (Kolman et al., 2019; Ramón-Llin et al., 2020; Sánchez-Alcaraz et al., 2020; Valldecabres et al., 2020). However, more research is needed in TT to describe the indicators involved during play and their relationship to performance (Djokić, 2006; Munivrana et al., 2015), and research involving women is practically non-existent. Undoubtedly, the results in TT competitions will be affected by physical fitness, physiological response, the metabolism involved or different psychological variables. However, technique and tactics can be considered in TT as one of the key aspects of performance, without underestimating others of great interest such as perception and decision, as they seem to have a direct effect on the results obtained during the competition (Huang et al., 2021).

In TT, each game starts with a serve or service and then the opponent immediately performs a motor response called serve-return. The serve and serve-return action is followed by a sequence of alternating strokes between the two players, ending when either player commits a fault (Wang, 2019). However, a play can also end with a direct service point (Reid et al., 2010), either because it is not returned correctly by the receiver or because of an error in the execution of the serve.

The service technique is considered one of the most critical performance indicators in racket sports (Ramón-Llin et al., 2021) as it is the action that starts each game (Katsikadelis et al., 2010). In the case of TT, the serve has evolved from being simply how the ball was put into space over a particular area, thus allowing game to begin, to become today a fundamental technical-tactical element that is essential to obtain a direct point (Pradas et al., 2009), as the serve is considered a technical action that can provide a tactical advantage.

The serve in TT is the only unforced technique during play (Yu et al., 2007). The TT serve action has infinite possibilities of execution, depending on the player’s level of skill and mastery of this technique, always within the legal limits allowed by the game’s rules. TT players specialise in this technical element as a basis for developing their own game, automating certain tactics depending on the type of serve. An infinite number of serves depends on the different characteristics of the playing materials and the player’s creativity in combining the different rotations that can be applied to the ball. In this sense, the serves in TT can be classified according to: (1) the distance of the bounce of the ball (close to the net, intermediate and close to the baseline); (2) the bounce zone of the ball (centre of the table, backhand zone and forehand zone); (3) the type of spin or rotation involved (slice, topspin, lateral or combined); and (4) the speed of the ball (fast or slow) (Pradas and Herrero, 2015).

Undoubtedly, a serve can greatly affect the opponent’s return and allow the player to gain an advantage to attack (Malagoli Lanzoni et al., 2014). In fact, it has been shown that in TT successful serves can lead to victory in a match (Katsikadelis et al., 2013). On the other hand, previous research has shown that the server’s tactical advantage is most significant in relatively short plays, up to the third stroke. In contrast, the probability of winning a play for the receiver increases after the fourth to sixth stroke (Malagoli Lanzoni et al., 2014). Thus, the serve advantage is lost as the length of the play increases. Although this pattern differs slightly between the sexes, the trend is very similar (Tamaki et al., 2017).

Another important game situation to consider in the sport of TT is the technical action of the return of the serve. An effective reception and return of the service allows the player to score a point outright or to enable the player to prepare well for game action to dominate subsequent attacks. Therefore, an effective serve and a successful reception and return are essential factors influencing the performance of a high-level TT player (Munivrana et al., 2015; Wang, 2019). Returning a serve correctly is undoubtedly the most difficult part of today’s TT technique. The return of the service is conditioned by the type of serve (spin, direction, speed, and location on the table), which makes it very difficult for the receiver to return. The correct reception and return of the serve is as important a technique as the serve itself, and a fundamental element in the development of tactics in this sport, as depending on the level of skill, it can allow a direct score to be obtained, prevent the opponent from taking the initiative in the game or provide an advantageous situation (Pradas et al., 2009). Among the most predominant techniques in serve and serve-return actions are the following: (a) Push, an interlocutory stroke imparting a back-spin effect to the ball; (b) Flip, an attacking stroke performed when the ball bounces close to the net; (c) Topspin, an attacking stroke actually imparting a topspin effect to the ball; (d) Block, a defensive stroke performed in response to a top in a passive fashion; (e) Lob, a defensive stroke performed when the player is far from the table, consisting of lifting the ball to a considerable height; (f) Smash, an attacking stroke characterised by a linear trajectory and no spin of the ball; and (g) Drive, an interlocutory stroke imparting no effect on the ball (Molodzoff, 2008).

Previous studies have qualified the importance of serving technique (Katsikadelis et al., 2013; Malagoli Lanzoni et al., 2014; Munivrana et al., 2015). However, studies that analyse the serve or return of the serve are scarce (Munivrana et al., 2015; Gómez et al., 2017; Wang et al., 2022), and even more so in high-level TT (Li and Li, 2008; Wang, 2019). Similarly, the serve and return of the serve analysis is practically non-existent in elite women TT players (Gómez et al., 2017). The differences between sexes in game dynamics have yet to be analysed in depth, and in this sense there is a great lack of knowledge about the technical-tactical situations of serve and return of the serve between men and women (Pradas et al., 2021). Therefore, the present study aimed to analyse the differences between sexes in the serve and return in elite TT players.

## 2. Materials and methods

### 2.1. Participants

In the present study, 24 men players (age: 25.3 ± 4.0 years; experience: 16.0 ± 4.1 years) and 24 women players (age: 22.3 ± 3.8 years; experiences: 13.2 ± 3.8 years) of different nationalities participated voluntarily.

The research was approved by the Clinical Research Ethics Committee of the Department of Health and Consumption of the Government of Aragon with file number 19/2010. All participants were informed of the procedures to be followed during the research and signed the consent form.

In order to participate in the research, subjects had to meet the following criteria: (a) belong to the senior category; (b) hold a federative licence from the Royal Spanish Table Tennis Federation; (c) play in the men’s or women’s first division of TT; (d) have at least 10 years’ experience; and (e) occupy at least 30th place in the men’s or women’s ranking in the Royal Spanish Table Tennis Federation senior category.

### 2.2. Study design

The present study was conducted in Seville (Spain) during the development of the matches corresponding to the final phases of two competitions, the Spanish Absolute Championship and the Spanish International Open. A total of 24 matches were studied (*n* = 12 males and *n* = 12 females). A total of 1,177 plays and 5,319 strokes in the men’s competition and 950 plays and 5,097 strokes in the women’s competition were analysed.

### 2.3. Analysis of technical-tactical actions

**Figure 1:**
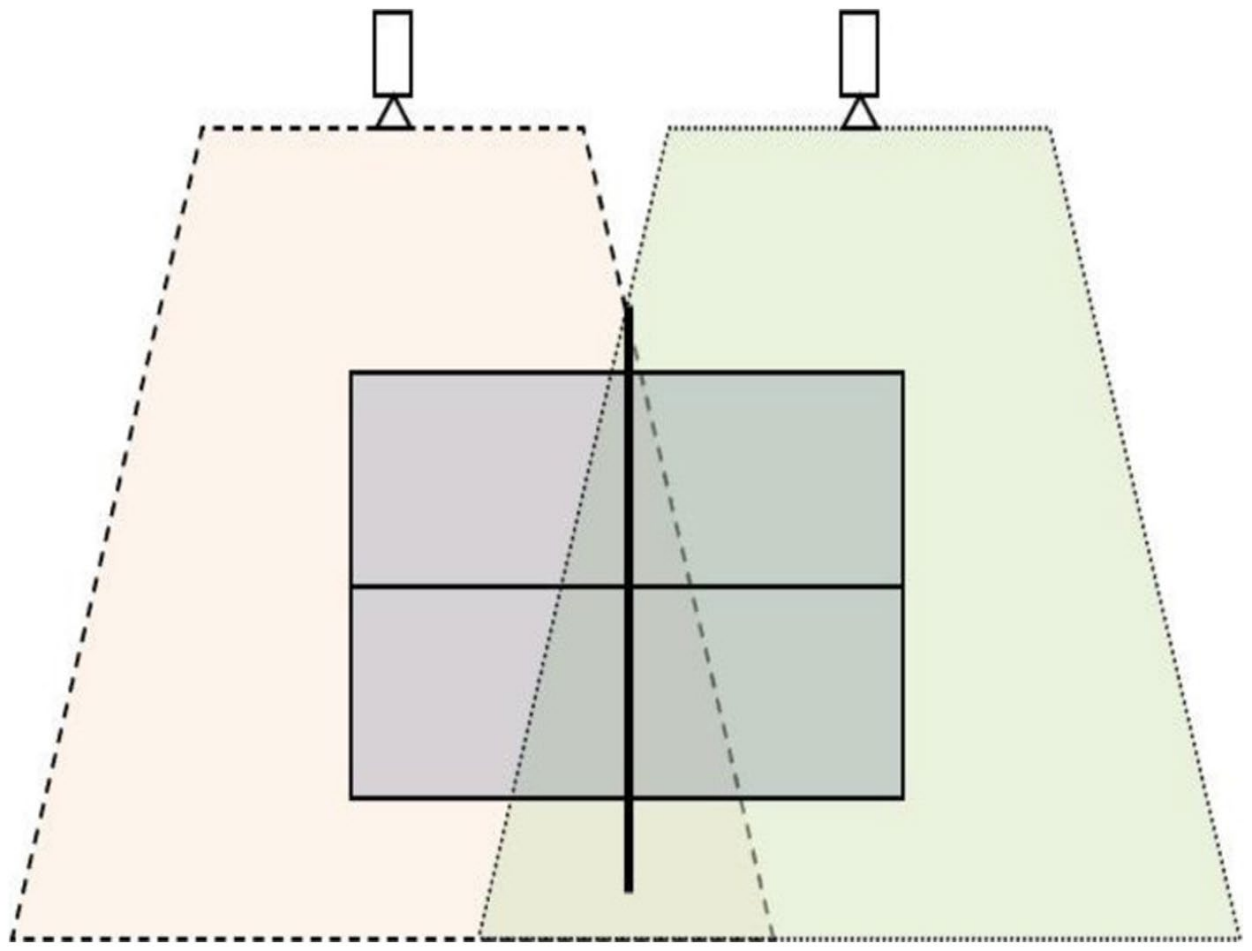
Competition recording protocol from an elevated side view (Pradas et al., 2021).

The matches were recorded using four Sony HDR-CX300E video cameras (Sony, Japan), placed on the sides of the tables at a minimum distance of 3 m and raised to a height of 2.5 m on telescopic supports (Manfrotto, 007 U, Italy). The games were recorded with a shutter speed of 1/500 s. Each camera filmed half a table from an elevated side view (Figure 1), obtaining two records of the game actions performed by each player.

**Figure 2:**
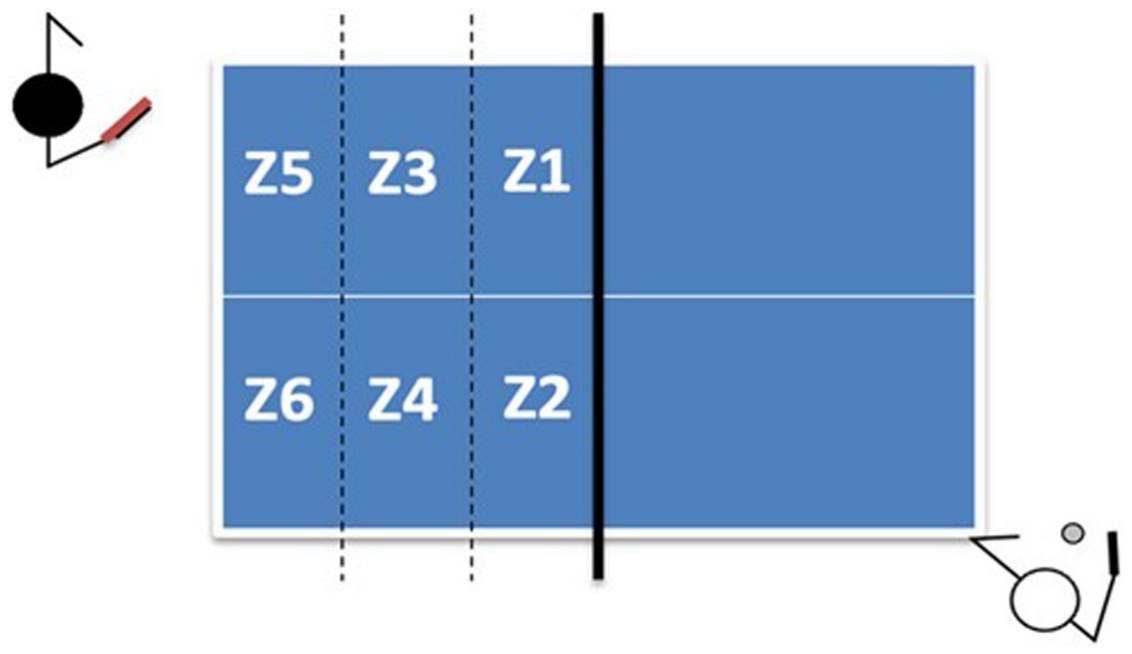
Distribution of the playing table into six zones. Zenithally view of the playing areas.

The playing surface was divided into six zones (each side), with a reference system placed on it, recorded by the cameras before the start of the match (Figure 2).

**Figure 3:**
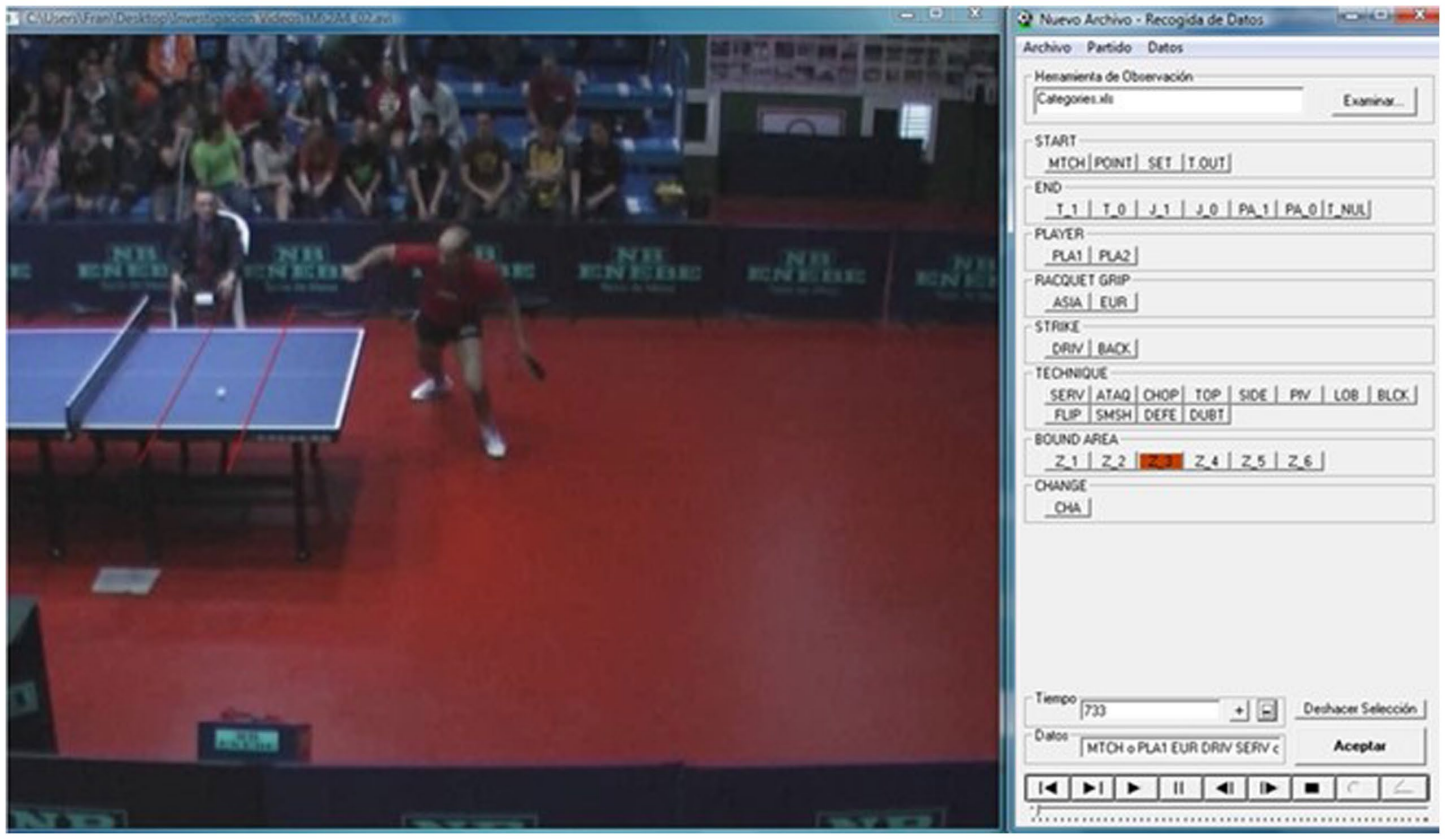
Example of data collection with the observation programme (Pradas et al., 2012).

After the recordings, a process of synchronisation of both cameras was carried out. The recorded matches were analysed using an observation tool validated for TT (Pradas et al., 2012), using the Match Vision Studio^©^ v. 3.0 programme (Perea et al., 2004), organised employing an *ad hoc* notational system that made it possible to study the times of game and break, as well as the different technical and tactical actions of the game (Figure 3).

Two TT coaches with level III of the maximum category of RFETM, experts in this sport, analysed the matches. The concordance analysis of the technical, tactical and time data obtained showed a Kappa index above 0.80 in all the variables analysed, considering the degree of agreement to be very high (Altman, 1991).

### 2.4. Statistical analysis

IBM® SPSS® Statistics Version 22 (IBM Corp., Armonk, NY, United States) was used for the analysis. Qualitative variables were expressed as percentages. The comparison between both sexes was made through contingency tables using the chi-square test.

Quantitative variables were expressed as mean and standard deviation and range. Normality was assessed using the Kolmogorov–Smirnov test and Levene’s test to evaluate homoscedasticity. For comparisons between sexes, the student’s t-test or the Mann–Whitney U test was used (according to normality and homoscedasticity). A value of *p* ≦ 0.05 was considered statistically significant.

## 3. Results

Table 1 presents the study participants’ technical characteristics (dominant hand, playing style, players grip and specific rubbers) according to sex. Differences between sexes were found in defensive style, grip style and backhand style (*p* < 0.05).

**Table 1:** Technical characteristics of the participants.

	Male (*n*)	%	Female (*n*)	%	χ^2^	*p*
Right-handed	17	70.8	20	83.3	1.061	0.300
Left-handed	7	20.2	4	16.7		
Defensive style	1	4.2	5	20.8	3.448	0.043
Offensive style	23	95.8	19	79.2		
Shake-hand grip	18	75	23	95.8	4.181	0.032
Pen-hold grip	6	25	1	4.2		
Pimples-in rubber (F)	21	87.5	23	95.8	1.091	0.296
Short pimples-out rubber (F)	3	12.5	1	4.2		
Pimples-in rubber (B)	22	91.8	18	75	5.567	0.039
Short pimples-out rubber (B)	1	4.1	1	4.1		
Long pimples-out rubber (B)	1	4.1	5	20.9		

Pearson’s Chi-square test. Values expressed in percentage F: forehand; B: backhand.

Table 2 shows the zone of serve on the table. Zones 1 and 2 were the most used by male players, while zone 5 was the predominant zone for female players (*p* < 0.05).

**Table 2:** Table serve zone.

	Male	Range	Female	Range
Zone 1 (*n*) +	9.17 ± 5.34	0–18	1.92 ± 3.06**	0–13
Zone 2 (*n*) −	12.17 ± 11.91	1–45	2.17 ± 2.12**	0–5
Zone 3 (*n*) −	38.42 ± 15.36	12–62	33.67 ± 17.35	17–70
Zone 4 (*n*) −	32.42 ± 20.71	5–71	28.17 ± 9.26	12–41
Zone 5 (*n*) +	4.25 ± 2.37	0–8	9.08 ± 3.89**	3–16
Zone 6 (*n*) +	1.67 ± 1.43	0–5	4.17 ± 5.42	0–20

**p < 0.01 male vs female differences; −: student’s *t*-test; +: non-parametric Mann–Whitney U test; values expressed as means ± standard deviation.

Tables 3, 4 shows the return of the serve overall and by ball placement zone in the table. In Table 3 there were differences between sexes in the total number of strokes. In Table 4, firstly, the return of the serve with push in zones 1 and 2 were predominant in male, while the return of the serve in zone 5 was more frequent in female (*p* < 0.05). On the other hand, concerning the return of the serve with the flip technique, zones 1, 3, and 4 were more frequent in male (*p* < 0.05). As for the return of the service with the topspin technique, there were differences in zone 3, being more frequent in male (*p* < 0.05). Finally, in the return of the serve push or chop in zones 3, 4, 5, and 6 were more frequent in female players (*p* < 0.05).

**Table 3:** Total serve-return types by sex.

	Male	%	Female	%	χ^2^	*p*
Push (*n*)	623	57.16	569	67.18	200.73	<0.001
Flip (*n*)	226	20.73	73	8.62		
Topspin (*n*)	207	18.99	105	12.40		
Block (*n*)	4	0.37	1	0.12		
Lob (*n*)	1	0.09	0	0.00		
Drive (*n*)	28	2.57	36	3.42		
Smash (*n*)	1	0.09	0	0.00		
Chop (*n*)	0	0.00	66	8.26		

Pearson’s Chi-square test. Values expressed in total strokes and percentage.

**Table 4:** Return of the service according ball placement zone.

	Male	Range	Female	Range
Reception with push				
Zone 1 (*n*)	6.58 ± 3.19	0–13	1.67 ± 3.11**	0–11
Zone 2 (*n*)	7.17 ± 4.95	1–18	1.92 ± 1.78**	0–4
Zone 3 (*n*)	19.83 ± 12.96	2–37	23.58 ± 14.13	7–50
Zone 4 (*n*)	18.08 ± 11.85	2–37	19.42 ± 8.43	9–30
Zone 5 (*n*)	0.25 ± 0.62	0–2	2.58 ± 2.61**	0–8
Zone 6 (*n*)	0.00 ± 0.00	0–0	1.25 ± 3.13	0–11
Reception with flip				
Zone 1 (*n*)	2.25 ± 1.42	0–4	0.17 ± 0.57**	0–2
Zone 2 (*n*)	4.25 ± 7.32	0–26	0.25 ± 0.45	0–1
Zone 3 (*n*)	9.08 ± 4.75	3–16	3.25 ± 3.72**	0–12
Zone 4 (*n*)	7.50 ± 5.05	2–19	2.08 ± 2.27**	0–8
Zone 5 (*n*)	0.08 ± 0.28	0–1	0.00 ± 0.00	0–0
Zone 6 (*n*)	0.00 ± 0.00	0–0	0.25 ± 0.62	0–2
Reception with topspin				
Zone 1 (*n*)	0.25 ± 0.86	0–3	0.00 ± 0.00	0–0
Zone 2 (*n*)	0.58 ± 0.79	0–2	0.00 ± 0.00	0–0
Zone 3 (*n*)	8.50 ± 3.82	3–18	2.83 ± 3.29**	0–12
Zone 4 (*n*)	6.25 ± 5.81	0–19	4.17 ± 3.51	1–13
Zone 5 (*n*)	2.17 ± 1.58	0–4	3.25 ± 3.19	0–12
Zone 6 (*n*)	1.08 ± 0.90	0–3	1.67 ± 1.92	0–6
Reception with block				
Zone 4 (*n*)	0.00 ± 0.00	0–0	0.08 ± 0.28	0–1
Zone 5 (*n*)	0.17 ± 0.57	0–2	0.00 ± 0.00	0–0
Zone 6 (*n*)	0.17 ± 0.38	0–2	0.00 ± 0.00	0–0
Reception with lob				
Zone 6 (*n*)	0.08 ± 0.28	0–1	0.00 ± 0.00	0–0
Reception with drive				
Zone 3 (*n*)	0.75 ± 1.21	0–9	0.50 ± 1.24	0–6
Zone 4 (*n*)	0.25 ± 0.62	0–3	1.08 ± 2.31	0–13
Zone 5 (*n*)	1.17 ± 1.74	0–14	0.58 ± 0.99	0–7
Zone 6 (*n*)	0.17 ± 0.38	0–2	0.25 ± 0.62	0–3
Reception with smash				
Zone 6 (*n*)	0.08 ± 0.28	0–1	0.00 ± 0.00	0–0
Reception with chop				
Zone 3 (*n*)	0.00 ± 0.00	0–0	2.75 ± 4.59**	0–33
Zone 4 (*n*)	0.00 ± 0.00	0–0	0.33 ± 0.88*	0–4
Zone 5 (*n*)	0.00 ± 0.00	0–0	2.00 ± 2.79**	0–24
Zone 6 (*n*)	0.00 ± 0.00	0–0	0.75 ± 1.28*	0–9

*p < 0.05; **p < 0.01 male and female differences. Student’s *t*-test. Values expressed as means ± standard deviation.

## 4. Discussion

In TT, the technical actions of the serve and return of the service are two of the most significant factors that can affect the result of TT match (Katsikadelis et al., 2013). The tactical implication of these two technical elements of the game is very important as they will define the development of game. Considering the critical relevance of these technical-tactical aspects in TT competition, the present study aimed to analyse the differences between sexes in the location of the service zone and the serve-return performed in elite players.

The results obtained in different investigations indicate that in order to win a TT match it is necessary to master two variables with great mastery and perfection, an excellent serving technique, and to possess the ability to execute a good technique for receiving and returning the ball, which is considered to be the game actions that make the difference between winners and losers in this sport (Djokić et al., 2020). On the other hand, as has been reported in previous studies (Munivrana et al., 2015; Pradas et al., 2021, 2022), there are sex differences in game structure, playing time and physical, physiological and metabolic demands. These factors cause differences in the development and application of technical-tactical actions.

Regarding the technical action of the serve, the results obtained in this research indicate to the existence of important differences between sexes in terms of preferences for the zone of the serve. Malagoli Lanzoni et al. (2014) reported that the serve plays a key role from a tactical point of view: (i) avoiding the opponent’s attack and (ii) allowing the server to attack the ball from the rest. Men mostly use the zones closer to the net while women do the opposite, using the areas closer to the baseline of the table, especially the opponent’s backhand area. This behaviour indicates that at a tactical level the men try to make their serve difficult or cancel out the opponent’s attack, while the women, on the contrary, prefer the opponent to initiate offensive actions immediately in order to develop a more counter-offensive game. These results are similar to those described for international cadet and junior (Mulloy et al., 2014) and senior top-level (Malagoli Lanzoni et al., 2014), with no data available for senior elite female players. However, in a study conducted on cadet greek female players, the young players chose as their main option serves over areas far from the net (Nikolakakis et al., 2021), and these results coincide with those obtained in this study for the female players. As can be seen, in both sexes the players avoid sending the ball to the right corner of the opponent’s table, i.e., zone 6 (for right-handed players), from which the attack for the receiver is easier to make and more dangerous for the server, thus preventing the receiver from taking the initiative to attack (Katsikadelis et al., 2013).

Players who win matches score more points when serving compared to losing players (Djokic, 2002; Katsikadelis et al., 2013). In this sense, according to Yu et al. (2007) when a player serves and then attacks they obtain a higher rate of points compared to the case when they attack after receiving the opponent’s serve.

Previous research has compared the server and return service characteristics of TT at the London (2012) and Rio (2016) Olympic Games, with significant changes observed in the percentage of the type of stroke used, in particular with the topspin and flip technique (Wang, 2019). The first decreased from 42.6% to 24.3%, while the latter increased from 5.1% to 16.6%. These data indicate that the players, when performing the serve technique, direct them towards areas close to the net, thus forcing the return of the service to perform either a defensive push or offensive flip technique, avoiding the service close to the baseline and preventing the return of the service from using the topspin technique, considered in this sport as the most offensive.

Different studies indicate that the push technique and the flip are the most frequent return of the serve actions in men’s competition (Malagoli Lanzoni et al., 2014), in line with the findings of this research. No current research has been found with which to compare the tactical indicators corresponding to the direction and zone of play, as the researchers applied different notational systems to the one used in this study (Kahn et al., 2004; Wu and Escobar-Vargas, 2007), so the results obtained for these tactical actions cannot be discussed. Recent studies on the location of the serve in elite players show that men direct their serves towards the central areas of the table, similar to that found in this study (Djokić et al., 2020).

Regarding the type of technical action by return of service and the area of game in which this tactical action is located, (Djokić, 2006) indicates out that it is very diverse, and that it depends on different variables, such as the area where the serve bounces, the style of play developed by the player, and the type of coating used on the racket. In this research, only the serve-return zone has been considered, so it cannot be compared with other reference studies. No current research has been found that describes these tactical indicators in the women’s game.

The present study is not without limitations: (i) the small number of participants; (ii) the style of play developed was not taken into consideration; (iii) the type of playing materials used was not taken into consideration; (iv) the lateral dominance of the players was not considered; and (v) psychological factors were not considered in this study. It is true that the stress generated during competition can affect the technical and tactical actions studied. Likewise, the need for more information on this topic makes it difficult to discuss the results. However, this fact allows the present manuscript to be novel in the area. For future research it could be interesting to analyse the above technical-tactical aspects in men’s, women’s and mixed doubles where there is a limitation and obligation to serve diagonally to the right.

In conclusion, there are sex differences in serve and return of the service actions during a match in elite TT players. Regarding the serve, male players preferentially use zones 1 and 2, while female players select zone 5 to a greater extent. Regarding the return of the service, there are differences between sexes according to the zone and the serve-return technique used. The results of this research provide valuable information on the predominant actions according to sex to choose the most appropriate system of play for TT players.

## Data availability statement

The original contributions presented in the study are included in the article/supplementary material, further inquiries can be directed to the corresponding author.

## Ethics statement

The research was approved by the Clinical Research Ethics Committee of the Department of Health and Consumption of the Government of Aragon with file number 19/2010. All participants were informed of the procedures to be followed during the research and signed the consent form.

## Author contributions

FP and CC: conceptualization. FP and LC: methodology and writing—review and editing. VT-R: formal analysis and data curation. FP, CC, and LC: investigation. VT-R and FP: writing—original draft preparation. FP: funding. All authors contributed to the article and approved the submitted version.

## Funding

This publication was financed from the Consejo Superior de Deportes to Research Project 10/UPB10/10 “TEMENOT. Studying the sport performance of top table tennis players by computerized notational analysis”. In addition, this publication has been supported by public funds from de Dirección General de Investigación e Innovación del Gobierno de Aragón to the ENFYRED researchgroup.

## Conflict of interest

The authors declare that the research was conducted in the absence of any commercial or financial relationships that could be construed as a potential conflict of interest.

## Publisher’s note

All claims expressed in this article are solely those of the authors and do not necessarily represent those of their affiliated organizations, or those of the publisher, the editors and the reviewers. Any product that may be evaluated in this article, or claim that may be made by its manufacturer, is not guaranteed or endorsed by the publisher.

## Abbreviation

TT: table tennis.
